# US Pilot Study of Lumbar to Sacral Nerve Rerouting to Restore Voiding and Bowel Function in Spina Bifida: 3-Year Experience

**DOI:** 10.1155/2014/863209

**Published:** 2014-06-02

**Authors:** Kenneth M. Peters, Holly Gilmer, Kevin Feber, Benjamin J. Girdler, William Nantau, Gary Trock, Kim A. Killinger, Judith A. Boura

**Affiliations:** ^1^Beaumont Health System, Royal Oak, MI 48073, USA; ^2^Oakland University William Beaumont School of Medicine, Rochester, MI 48309, USA; ^3^Urology Center of the Rockies, Fort Collins, CO 80528, USA

## Abstract

*Objective*. To report our experience with creating a skin-central nervous system-bladder reflex arc with intradural lumbar to sacral motor root microanastomosis to restore bladder/bowel function in spina bifida patients. *Methods*. Urinary/bowel changes from baseline to three years were evaluated with questionnaires, voiding diaries, urodynamics (UDS), and renal function studies. Treatment response was defined as CIC ≤ once/day with stable renal function, voiding efficiency > 50%, and no worsening of motor function. *Results*. Of 13 subjects (9 female, median age 8 years), 3 voided small amounts at baseline, one voided 200 cc (voiding efficiency 32%), 4/13 reported normal bowels, and 2/13 were continent of stool. Postoperatively, all had transient lower extremity weakness; one developed permanent foot drop. Over three years, renal function remained stable and mean maximum cystometric capacity (MCC) increased (*P* = 0.0135). In the 10 that returned at 3 years, 7 were treatment responders and 9 had discontinued antimuscarinics, but most still leaked urine. Only 2/8 with baseline neurogenic detrusor overactivity (NDO) still had NDO, all 3 with compliance <10 mL/cm H_2_O had normalized, 7/10 considered their bowels normal, 5/10 were continent of stool, and 8/10 would undergo the procedure again. *Conclusion*. Lumbar to sacral nerve rerouting can improve elimination in spina bifida patients. This trial is registered with ClinicalTrials.gov NCT00378664.

## 1. Introduction

The concept of joining healthy nerves to injured nerves to improve function is not new. In 1828, Flourens showed that the distal end of one transected nerve could make functional union with the proximal end of another nerve [1]. Subsequently, Langley and colleagues demonstrated that cholinergic preganglionic autonomic nerves can make connections to striated muscle [2]. In 1907, Kilvington proposed that the neurogenic urinary bladder might be reinnervated by somatic nerves to improve function. He even attempted this in one patient but was unsuccessful [3].

During the past century, numerous studies have been conducted in animals and humans to evaluate the possible functional consequences of bladder reinnervation using somatic-autonomic nerve cross union. In 1967, Carlsson and Sundin reported on a four-year-old spina bifida patient who underwent rerouting of the thoracic 10-11 ventral (motor) roots to S1-S2 ventral roots. After eight months of recovery, reflex micturition and bladder sensation appeared [4]. Despite this previous interest in nerve rerouting to reinnervate the neurogenic bladder, it was Xiao and Godec who further pursued this concept [5]. Studies were first done in animals, confirming that the bladder could be reinnervated by a somatic nerve and that reflex micturition could occur [5, 6]. This was followed by reports of some early clinical success in humans with spina bifida [7].

We conducted the first North American pilot trial to evaluate the impact of nerve rerouting on urinary and bowel function in spina bifida. The primary intent was to explore the procedure's safety and, secondly, to assess clinical outcomes over 3 years. We have previously reported one-year outcomes in the first 9 enrolled patients. The purpose of this paper is to describe our experience and the three-year outcomes in all 13 patients who had the procedure as part of our exploratory pilot study performed in the United States.

## 2. Materials and Methods

### 2.1. Study Design and Sample

A pretest posttest design was used with a convenience sample. After the study received Institutional Review Board approval, informed consent/assent was obtained from potential subjects and/or their parents. Male and females, age six years and older, with spina bifida (myelomeningocele or lipomeningocele) and neurogenic bladder confirmed during urodynamics studies (UDS), using clean intermittent catheterization (CIC) for bladder management, and with normal renal ultrasound, BUN, and creatinine were enrolled. Patients with vesicoureteric reflux ≥ grade two, strictures, or bladder capacity <100 mL were excluded. Baseline neuroimaging, neurological exam, neurophysiologic testing, and computerized UDS were performed. Uroflow with measurement of post-void residual and cutaneous stimulation of the anticipated operative dermatome to test for reflex detrusor contraction were not attempted in the first nine cases at baseline but were assessed in the last four subjects that were enrolled. Questionnaires were developed to assess changes in urinary function since no appropriate instruments for use in children with spina bifida were found in the literature. Bowel function was assessed with a questionnaire developed for use in spinal cord injured patients (Burwood Bowel Questionnaire) [8]. Voiding diaries and Global Response Assessments (GRA; patients rate changes since treatment on a seven-point scale of “markedly worse” to “markedly better”) were also used to assess outcomes.

### 2.2. Description of Procedure

We attempted to enroll patients that were similar in characteristics to those reported by Xiao and colleagues [7]. Dr. Xiao provided assistance to our team of urologists to ensure consistency with his technique, and a pediatric neurosurgeon that had been recently recruited to our institution joined the team for the last four cases. Subjects underwent unilateral rerouting of the ventral lumbar root to the sacral roots through a limited laminectomy. The procedure has been previously described [9]. Briefly, needle electromyography (EMG) electrodes were inserted in the lower extremities for continuous intraoperative neurophysiological monitoring. A limited laminectomy was performed at the level of the first intact spinous process. The lowest nerve root that exhibited a reproducible muscle EMG response with intraoperative stimulation was chosen as the donor nerve. The ventral root of the donor nerve was identified, and an attempt was made to split the donor nerve longitudinally to keep a portion intact and decrease the likelihood of foot drop. The distal stump of the donor ventral root was anastomosed to the proximal stump of the S3 ventral root.

### 2.3. Outcomes Definitions

Patients were followed closely for three years postoperatively with clinical and functional examinations and questionnaires. Clinical response (treatment success) was defined as voiding with at least 50% efficiency on uroflow, using CIC ≤ once per day (as recorded on voiding diaries) with stable renal ultrasound, BUN, and creatinine, and no worsening of motor function from baseline. Secondary outcomes included evidence of a new neural pathway to elicit voiding, demonstrated by reproducible (at least two) detrusor contractions during the same UDS of at least 10 cm/H_2_O rise in detrusor pressure (pdet) while performing cutaneous stimulation (scratching) of the operative dermatome. Other secondary outcomes were changes in bladder compliance (normal compliance >10 mL/cm H_2_O), neurogenic detrusor overactivity (NDO), and overall changes in urinary and bowel function on questionnaires. Given this small patient cohort, mostly descriptive statistics were performed. Repeated measures analyses were completed for change in maximum cystometric capacity (MCC) over time. The MCC was included as the dependent variable with time as the independent variable in a general linear model adjusting for repeated observations per patient.

## 3. Results

Thirteen patients (nine females and four males) were enrolled. Median age was eight years old (range was six to 37 years) and the majority (*n* = 11) had overactive neurogenic bladders; two were underactive. All were ambulatory; eight required no assistive devices, one required ankle foot orthosis (AFO), and five had leg braces (two also used crutches). Eight patients had their spinal defect closed at birth, three had intrauterine closure, and two had no cutaneous defect and no closure. Six patients had undergone previous cord detethering surgery.

During the perioperative period, two patients had increased wound drainage that resolved with prone positioning, but two others with suspected cerebral spinal fluid (CSF) leak required another surgical procedure at 25 and 37 days postoperatively. Twelve patients had transient lower extremity weakness that resolved by 12 months, and one that had intrauterine closure of her defect developed permanent foot drop after surgery. Renal ultrasounds and serum creatinines remained stable in all patients throughout the study.

At three years, three patients (including the one with permanent foot drop) did not return for follow-up and were considered nonresponders based on a lack of positive response at their two-year visit. In the remaining 10 subjects, even though only four were able to void at all at baseline, eight of 10 were voiding at three years (Table 1). However, using our definition of treatment success, nerve rerouting was successful in only seven of 13 (54%), since one other was only voiding with 47% efficiency. This patient had been a “responder” at two years but developed a tethered cord by the 3-year visit and was only voiding with 47% efficiency. Anecdotally, this patient continues to be followed at our center and is now voiding with >50% efficiency after undergoing rerelease. Although eight were voiding at three years, only four of eight patients reported that their urine stream was strong and all but one used Valsalva to initiate voiding (one scratched the operative dermatome to initiate voiding). The other nonresponders were two of the three that had intrauterine closure, our one adult subject (37-year-old), the one subject with foot drop, and another seven-year-old.

Pre- to postoperative changes in the entire sample are depicted in Table 1. A statistically significant increase in maximum cystometric capacity (MCC) was seen over time (*P* = 0.0135). At baseline, eight of 13 had neurogenic detrusor overactivity (NDO), but by three years only 2/8 with baseline NDO still had NDO (one patient that was not voiding and the one with retethering). By three years, all ten subjects were able to stop using antimuscarinics except for one nonvoiding patient with persistent NDO. Three of four patients with baseline compliance of <10 mL/cm H_2_O had normalized, and the fourth did not return for a three-year visit but had normalized at two years (Table 2). Two patients with normal compliance at baseline had compliance <10 mL/cm H_2_O at three years. One of these was found to have a symptomatic tethered cord at their three-year visit with worsening of bladder function and lower extremity spasticity. This patient had been voiding with >50% efficiency at 2 years but developed a tethered cord by his 3-year visit. He had rerelease of tethered cord, continues to be followed clinically at our center, and reportedly is again voiding similar to his 2-year study outcomes. The other had their three-year UDS at an outside institution near their home. This patient had an active UTI at their three-year study visit and UDS was deferred, which may have influenced calculated compliance. Even though eight of 13 had demonstrated reproducible, sustained detrusor contractions with cutaneous stimulation at 12 months, by three years only two still had reproducible contractions. Interestingly, the two patients that still had reproducible detrusor contractions used Valsalva to initiate voiding but one other patient without demonstrable reflex still used cutaneous stimulation to initiate voiding at three years.

Table 3 contains baseline and three-year data for each individual subject. All thirteen patients were incontinent of urine at baseline. In the 10 patients with three years of followup, only one of ten was dry and the rest had persistent stress incontinence. Five of ten reported at least some improvement in overall urinary function on the GRA but five reported no change even though three of these were considered treatment responders. Improvements in bowel function were also seen. At baseline, only four of 13 considered their bowels normal and two of 13 were continent of stool. At three years, seven of 10 considered their bowels normal, five of 10 were continent of stool, and five of 10 reported overall improvement in bowel function on the GRA. Other than one patient with persistent foot drop, no long-term complications were identified and eight of 10 subjects stated they would undergo the procedure again.

## 4. Discussion

This pilot trial represents the first North American study on lumbar to sacral nerve rerouting to restore bladder and bowel function in spina bifida. All of our patients were ambulatory preoperatively. Even though this was essential to assure a robust donor lumbar nerve to reroute to the sacral plexus, performing this procedure on the ambulatory patient poses increased risks. The majority of patients had transient weakness postoperatively but had returned to baseline function by one year. However, one subject developed persistent foot drop that affected the quality of her ambulation and, unfortunately, she also did not have any improvement in her bowel or bladder function. Although the cause of this is uncertain, this and one of two others with intrauterine closure of their defect did not achieve treatment success and it was felt by the surgeons that there was more scarring, making the surgery more difficult. Thus, prior intrauterine closure became exclusionary as the trial progressed. Since permanent foot drop was the most significant procedural risk identified, it is essential that patients considering this surgery are well informed of the potential risk.

Seven of thirteen subjects were considered clinical responders at three years, but one other might also be considered a responder based on his two-year data and subsequent clinical followup after a detethering procedure at three years. The three patients who did not return at three years were appropriately considered nonresponders based on their earlier visits that demonstrated no substantial clinical improvements. The other nonresponders were our 37-year-old and a seven-year-old; however the reasons for nonresponse are unclear, since the small sample size did not allow for examining predictors of treatment response. Overall, the remaining children did have clinically significant improvements in their bowel and bladder function.

It is unclear whether the increase in MCC resulted from rerouting, physical growth, or resolution of NDO. Changes in sensation were difficult to objectively assess; however, as we have previously reported [9], improvements in patients' ability to correctly identify a full bladder and the need to void during urodynamic testing were seen. Also, even though we did not assess urethral sphincter innervation/activity, there was no clinical evidence of postoperative detrusor-sphincter dyssynergia and renal function remained stable. Some subjects reported greater improvements in bowel function than bladder function, which may have positively influenced their overall satisfaction with the procedure. Fecal incontinence resolved in three patients who were incontinent of stool at baseline and three of four patients that did not consider their bowels normal at baseline reported normalization at three years. Only one patient was dry, so stress incontinence still remained a problem for most patients who were off CIC even though, overall, NDO had resolved.

Understanding the clinical significance of being able to demonstrate a cutaneous-to-bladder reflex on UDS is difficult. At one year, eight of thirteen subjects had a reproducible reflex. Even though finding this reflex provided some evidence that the bladder had been reinnervated, its presence did not always agree with the clinical response. By three years, this reflex was only demonstrated in two patients yet most (including these two) could void efficiently using some degree of Valsalva. Understanding the emergence and apparent suppression of this reflex over time is challenging. Either the bladder is no longer innervated by the somatic nerve, which seems unlikely given the clinical improvements, or the reflex becomes suppressed. Perhaps over time there is reconfiguration of the micturition centers in the brain and as the child learns to void independently the brain suppresses this reflex as seen in toilet training. It would be fascinating to perform functional magnetic resonance imaging (fMRI) of the brain before and after nerve rerouting to see what occurs with stimulation of this reflex compared to patients who recover sensation and are able to void without reflex stimulation. It would also be interesting to examine the relationships between cutaneous stimulation and rectal activity and improved bladder sensation and the emergence/disappearance of the skin-bladder reflex.

There are several important limitations to this pilot trial. First, there were no control subjects. Comparing subjects to a cohort of patients with similar age and bladder and bowel dysfunction undergoing maximum clinical care would strengthen the findings. However, since myelodysplastic lesions are dynamic throughout childhood, controls would be susceptible to the negative effects of growth spurts or tethered cord syndrome, which can occur in approximately one third of patients [10]. Another potential limitation is that, as some have critically suggested, outcomes may be related more to coincidental cord detethering during the procedure than nerve rerouting. The likelihood that outcomes of this study resulted solely from detethering is limited for two reasons. First, since all patients had stable motor, bladder, and bowel function at enrollment no clinical evidence of a tethered cord existed. Secondly, improvements in UDS parameters and/or clinical outcomes appear as early as four weeks [11] to five months [12] after detethering, yet at six months a number of our patients reported worsening of incontinence [9] prior to seeing some improvements at 12 months. This delay in improvement was not consistent with what would be expected after detethering and suggests that perhaps with time reinnervation and neural regeneration had taken place. Despite attempting to identify the appropriate nerve roots while minimizing dissection of the cord structures, some coincidental detethering likely occurred but its impact on outcomes is difficult to determine.

Several important insights were gained during this pilot trial that will benefit future studies. First, this procedure had never been performed on patients that had intrauterine closure of their defect. Since there was no prior experience to guide us in dealing with the significant anatomical challenges that arose we subsequently added prior intrauterine closure as an exclusionary criterion. Second, despite successful outcomes from nerve rerouting, future symptomatic cord tethering can occur in spina bifida. This was seen in a child who at two years was a clinical responder but, at year three, developed symptomatic cord tethering with lower extremity spasticity and worsening of bladder and bowel function. If detethering is needed, it is imperative that the neurosurgeon repairing the tethering identifies and spares the previous rerouting anastomosis to recover bladder and bowel function. We also did not get robust data on urethral sphincter activity. Future studies should objectively assess urethral and anal sphincter innervation with needle EMG in order to obtain evidence of neural regeneration. Video urodynamics would also be useful to gain a better understanding of the bladder neck and sphincter activity during voiding as well as assess for reflux. Also, in the first nine patients we did not attempt uroflow at baseline or test to see if cutaneous stimulation of the anticipated operative dermatome would elicit a detrusor contraction prior to surgery. Future studies of nerve rerouting should include these assessments at baseline, as well as urethral sphincter EMG, pad weights, anal manometry, and objective measurements bladder sensation. In the near future we plan to incorporate some of these measures into an extended follow-up study of our cohort of 13 subjects that had nerve rerouting at our institution.

As with any procedure it is important to balance the risks with the benefits. This is a very complex patient population with life-long challenges. Some patients are bothered by urinary retention, incontinence, urinary tract infections, social issues, constipation, fecal incontinence, mobility issues, or a combination of all of these. Which of these problems need to improve in order for the procedure to be considered successful? Aside from the definition of success being highly individualized from the patient's perspective, we have struggled with our definition of clinical success because absolute normalization of these functions is not a realistic goal in this patient population. In the United States, neurogenic bladder and bowel can be safely controlled with the use of intermittent catheterization, antimuscarinics, augmentation, and a bowel regimen. Thus, improvement in quality of life with minimal risk could be considered a successful outcome. However, in underdeveloped countries where intermittent catheterization and antimuscarinics are not readily available, neurogenic bladder leads to renal failure and death. The definition of success in these countries is likely quite different and less driven by quality of life and more by survival.

## 5. Conclusions

This pilot trial demonstrated that bladder and bowel function in patients with spina bifida can improve after lumbar to sacral nerve rerouting. It is important to conduct further studies employing lessons learned from this trial and carefully obtain consent of patients prior to offering this procedure. Patients and their families need to understand that one cannot predict up front who will respond positively and that there is a potential for permanent foot drop that can adversely affect ambulation. Even though more data are needed to fully understand the impact of this procedure, nerve rerouting has the potential to change how patients with neurogenic bladder are managed.

## Figures and Tables

**Table 1:** Summary of changes over time.

	Baseline		One year		Two years		Three years	
	*N*	*N* (%)	*N*	*N* (%)	*N*	*N* (%)
NDO	13	8 (61.5)	13	2 (15.4)	13	5 (38.5)	10	2 (20)
Able to void	13	4 (30.8)	13	9 (69.2)	13	10 (76.9)	10	8 (80)
On CIC	13	13 (100)	13	10 (76.9)	13	10 (76.9)	10	2 (20)
UI	13	13 (100)	13	13 (100)	13	12 (92.3)	10	9 (90)
Cutaneous bladder reflex present	4	0	13	8 (61.5)	13	6 (46.2)	9	2 (22.2)
Consider bowels normal	13	4 (30.8)	13	3 (23.1)	13	5 (38.5)	10	7 (70)
*Improved urinary function			13	7 (53.9)	13	9 (69.2)	10	5 (50)
Continent of stool	13	2 (15.4)	13	4 (30.8)	13	6 (46.2)	10	5 (50)
*Improved bowel function			13	8 (61.5)	13	7 (53.9)	10	5 (50)

	*N*	Mean ± SD (median)	*N*	Mean ± SD (median)	*N*	Mean ± SD (median)	*N*	Mean ± SD (median)

MCC	13	213.46 ± 84.68 (200)	13	250.77 ± 91.29 (230)	13	249.38 ± 60.87 (246)	10	291.1 ± 94.04 (284)
Voided volume	13	22.62 ± 54.91 (0)	13	118.92 ± 125.24 (70)	13	137.31 ± 130.49 (100)	10	211.9 ± 133.58 (242.5)
PVR	4	231.75 ± 154.56 (208.5)	12	126.25 ± 120.94 (100)	13	122.31 ± 108.10 (125)	10	131.40 ± 97.05 (132.50)

NDO: neurogenic detrusor overactivity; CIC: clean intermittent catheterization >1x/day; UI: urinary incontinence; MCC: maximum cystometric capacity; PVR: post-void residual.

*Slightly, moderately, or markedly improved response on Global Response Assessments (GRA).

**Table 2:** Compliance (mL/cm H_2_O).

Study ID	Baseline	Three years
1	6.6	34.3
2	14.3	ND
3	8.3	28.4
4	28.6	23.2
5	24	14.8
6	17	71.3
7	17.4	ND
8	9.4	21.2
9	19.5	37.7
10	162	48.9
11	14	5.6
12	8	ND
13*	22	5.1

ND: no data.

*Retethered.

**Table 3:** Individual patient data.

Pt. number	Age (Yrs)	On CIC		Using antimuscarinics		NDO		Voided volume^†^		Voiding efficiency (%)		MCC (cc)		Consider bowels normal		Continent of stool		Change in bowel function (GRA)	Treatment responder
		Baseline	3 Yr.	Baseline	3 Yr.	Baseline	3 Yr.	Baseline	3 Yr.	Baseline	3 Yr.	Baseline	3 Yr.	Baseline	3 Yr.	Baseline	3 Yr.	3 Yr.	3 Yr.
1	6	Yes	No	No	No	No	No	0	260	∗	84	252	240	No	Yes	No	No	Mildly worse	Yes
3	13	Yes	No	Yes	No	Yes	No	0	300	∗	63	165	342	No	No	No	Yes	Moderately improved	Yes
4	7	Yes	No	No	No	Yes	No	0	270	∗	66	200	316	No	Yes	No	Yes	Markedly improved	Yes
5	6	Yes	No	Yes	No	No	No	0	75	∗	60	48	134	Yes	Yes	No	No	Slightly improved	Yes
6	17	Yes	No	No	No	No	No	0	360	∗	95	350	428	Yes	Yes	Yes	Yes	No change	Yes
8	8	Yes	No	Yes	No	Yes	No	22.5	225	∗	64	189	212	Yes	Yes	No	Yes	Markedly improved	Yes
10	15	Yes	No	Yes	No	Yes	No	200	380	32	97	324	391	No	Yes	Yes	Yes	No change	Yes
2	7	Yes	∗	Yes	∗	Yes	∗	0	∗	∗	∗	200	∗	No	∗	No	∗	∗	No
7	8	Yes	∗	Yes	∗	Yes	∗	0	∗	∗	∗	226	∗	No	∗	No	∗	∗	No
9	37	Yes	Yes	No	Yes	Yes	Yes	26.6	30	∗	8	269	377	No	Yes	No	No	Slightly improved	No
11	7	Yes	Yes	Yes	No	No	No	5	0	3	0	167	219	No	No	No	No	No change	No
12	6	Yes	∗	No	∗	No	∗	0	∗	0	∗	101	∗	Yes	∗	No	∗	∗	No
13^‡^	11	Yes	No	No	No	Yes	Yes	40	219	13.5	47	284	252	No	No	No	No	No change	No

MCC: maximum cystometric capacity; NDO: neurogenic detrusor overactivity; CIC: clean intermittent catheterization >1x/day; UI: urinary incontinence; PVR: post-void residual; GRA: Global Response Assessment.

^†^On voiding diary at baseline for subjects 1–9; on uroflow at 3 yr.

*Missing data.

^‡^Tethered cord at 3 yr. visit.

## Abbreviations

UDS: Urodynamic studies
NDO: Neurogenic detrusor overactivity
MCC: Maximum cystometric capacity
CIC: Clean intermittent catheterization
EMG: Electromyography
GRA: Global response assessment
AFO: Ankle foot orthosis.
